# Design and synthesis of bioactive molecules

**DOI:** 10.3762/bjoc.22.112

**Published:** 2026-10-07

**Authors:** Fengzhi Zhang, David R Spring

**Affiliations:** 1 School of Pharmacy, Hangzhou Medical College, Hangzhou, 310014, P. R. China; 2 Yusuf Hamied Department of Chemistry, University of Cambridge, Lensfield Road, CB2 1EW, Cambridge, United Kingdom

**Keywords:** bioactive molecules, design, synthesis

The design and synthesis of bioactive molecules remains a central pillar of modern chemical science, underpinning advances in drug discovery, chemical biology and molecular medicine. The ability to create molecules that modulate biological systems with precision continues to evolve rapidly, driven by innovations in synthetic methodology, molecular design and interdisciplinary integration. In this thematic issue, we present 8 Full Research Papers, 2 Letters, 2 Reviews and 1 Perspective, reflecting the breadth and emerging directions of this dynamic field.

A prominent theme is the growing importance of targeted protein modulation, particularly through bifunctional molecules. The development of an erdafitinib-derived degrader demonstrates selective degradation of FGFR2 and highlights the potential of targeted protein degradation to overcome resistance and off-target effects associated with conventional inhibitors [1]. Complementing this, the design of CAM4066-based PROTACs targeting CK2 illustrates how structure-guided linker installation can preserve binding interactions while enabling E3 ligase recruitment [2]. These advances are contextualised by a comprehensive Review on PROTACs, which emphasises their ability to achieve high selectivity within homologous protein families through event-driven pharmacology [3].

The issue also highlights advances in chemical biology tools and reactive probe development. A rhodium-catalysed strategy enables the connective synthesis of diverse reactive probes bearing S(VI) electrophilic warheads, expanding the toolkit for covalent probe design and target engagement [4]. In parallel, the development of silicon-rhodamine derivatives via a practical CO_2_-mediated carboxylation strategy provides efficient access to key intermediates for targeted imaging probes, facilitating applications in live-cell super-resolution microscopy [5]. Together with a work on nanogold-labelled HDAC probes [6], these studies demonstrate how synthetic innovation enables increasingly sophisticated interrogation of biological systems.

At the interface of peptide science and small-molecule design, a Perspective explores how generative artificial intelligence can transform peptide binders into small-molecule leads, spanning workflows from structure prediction to scaffold generation and candidate prioritisation [7]. This is complemented by experimental advances in peptide chemistry, including the efficient solid-phase synthesis and structural characterisation of segetalins A–H, J and K, which highlights the continued importance of synthetic access to structurally complex bioactive peptides [8]. A Review on site-specific labelling strategies further underscores the importance of selective and scalable modification of native peptides and proteins, with broad implications for chemical biology and therapeutic development [9].

The enduring influence of natural-product-inspired and bioactive scaffold synthesis is reflected in contributions exploring structure–activity relationships and biological evaluation. The synthesis of brassinosteroid analogues demonstrates how subtle structural modifications influence receptor binding, signalling pathways and biological responses [10]. Through the functional group splicing strategy, the design of a novel FXR/ASK1 dual-target modulator Z8 displays potential promise for the treatment of metabolic dysfunction-associated steatohepatitis (MASH) [11]. The synthesis and evaluation of glycidyl esters of phosphorus acids highlights the development of novel bioactive scaffolds with potential anticancer activity, supported by both biological assays and electrochemical studies [12]. In addition, a prodrug approach yielded the ʟ-alanine conjugate AKBA-Ala, which showed anti-inflammatory potency comparable to that of AKBA [13].

Across these contributions, several unifying themes emerge. First, there is a clear shift toward functional molecular design, where compounds are engineered not only to bind targets but to induce specific biological outcomes. Second, synthetic efficiency and practicality remain essential, with new methodologies enabling more rapid, scalable and sustainable access to functional molecules. Third, the integration of computational and experimental approaches, particularly through artificial intelligence, is reshaping how bioactive molecules are conceived and optimised.

We are grateful to all authors for their outstanding contributions and to the reviewers for their insightful evaluations. We also thank the editorial team for their support in assembling this thematic issue. We hope that this collection will serve not only as a snapshot of current advances but also as a source of inspiration for future developments in the design and synthesis of bioactive molecules.

As chemistry continues to converge with biology and data science, the opportunities for innovation are immense. The work presented in this issue reflects this momentum and highlights the central role of chemical synthesis in addressing complex biological challenges.

Fengzhi Zhang and David R. Spring

Hangzhou and Cambridge, September 2026

## Data Availability

Data sharing is not applicable as no new data was generated or analyzed in this study.
